# The Enigma of Substrate Recognition and Catalytic Efficiency of APE1-Like Enzymes

**DOI:** 10.3389/fcell.2021.617161

**Published:** 2021-03-26

**Authors:** Anastasiia T. Davletgildeeva, Alexander A. Ishchenko, Murat Saparbaev, Olga S. Fedorova, Nikita A. Kuznetsov

**Affiliations:** ^1^Institute of Chemical Biology and Fundamental Medicine, Siberian Branch of the Russian Academy of Sciences, Novosibirsk, Russia; ^2^Department of Natural Sciences, Novosibirsk State University, Novosibirsk, Russia; ^3^Group “Mechanisms of DNA Repair and Carcinogenesis”, Equipe Labellisée LIGUE 2016, CNRS UMR 9019, Université Paris-Saclay, Villejuif, France

**Keywords:** DNA repair, apurinic/apyrimidinic endonuclease, abasic site, target nucleotide recognition, pre-steady state kinetics

## Abstract

Despite significant achievements in the elucidation of the nature of protein-DNA contacts that control the specificity of nucleotide incision repair (NIR) by apurinic/apyrimidinic (AP) endonucleases, the question on how a given nucleotide is accommodated by the active site of the enzyme remains unanswered. Therefore, the main purpose of our study was to compare kinetics of conformational changes of three homologous APE1-like endonucleases (insect *Drosophila melanogaster* Rrp1, amphibian *Xenopus laevis* xAPE1, and fish *Danio rerio* zAPE1) during their interaction with various damaged DNA substrates, i.e., DNA containing an F-site (an uncleavable by DNA-glycosylases analog of an AP-site), 1,*N*^6^-ethenoadenosine (εA), 5,6-dihydrouridine (DHU), uridine (U), or the α-anomer of adenosine (αA). Pre-steady-state analysis of fluorescence time courses obtained for the interaction of the APE1-like enzymes with DNA substrates containing various lesions allowed us to outline a model of substrate recognition by this class of enzymes. It was found that the differences in rates of DNA substrates’ binding do not lead to significant differences in the cleavage efficiency of DNA containing a damaged base. The results suggest that the formation of enzyme–substrate complexes is not the key factor that limits enzyme turnover; the mechanisms of damage recognition and cleavage efficacy are related to fine conformational tuning inside the active site.

## Introduction

The maintenance of DNA integrity is ensured by repair enzymes that recognize, remove, and restore the structure of damaged DNA regions (Friedberg et al., 2006). One way to remove nonbulky damaged bases is the base excision repair (BER) pathway, which is initiated by numerous damage-specific DNA glycosylases (Gros et al., 2002; Fromme et al., 2004). The removal of damaged bases from DNA in the first step of BER is coupled to the second step (catalyzed by an AP endonuclease), intended to remove apurinic/apyrimidinic (AP) sites and 3′-blocking sugar phosphate groups by the hydrolysis of the phosphodiester bond on the 5′ side of the lesion (Demple and Sung, 2005; Li and Wilson, 2014). As the result of AP endonuclease action, the formation of a single-nucleotide gap with 3′-OH and 5′-phosphate end groups takes place, which is necessary for DNA template-directed incorporation of an intact nucleotide by DNA polymerase and DNA ligation of the strand break. It is also known that AP endonucleases can recognize not only AP-sites but also various damaged nucleotides such as 5,6-dihydrouridine (DHU), α-anomers of nucleotides, 1,*N*^6^-ethenoadenosine (εA), uracil (U), and other modified residues (Gros et al., 2004). In addition, AP endonucleases have 3′-5′-exonuclease (Chou and Cheng, 2003; Wong et al., 2003; Kuznetsova et al., 2018a) and endoribonuclease (Barzilay and Hickson, 1995; Berquist et al., 2008; Barnes et al., 2009; Kuznetsova et al., 2020) activities.

A major human AP endonuclease, human APE1 (hAPE1) is one of the most studied AP endonucleases. Indeed, multiple structural data (Gorman et al., 1997; Mol et al., 2000a,b; Beernink et al., 2001; Manvilla et al., 2013; Tsutakawa et al., 2013; Freudenthal et al., 2015), kinetic studies (Timofeyeva et al., 2009; Miroshnikova et al., 2016a,b; Alekseeva et al., 2019a), and a mutational analysis (Erzberger and Wilson, 1999; Alekseeva et al., 2020) have made it possible to identify the key stages of the interaction of hAPE1 with a damaged DNA harboring an AP site or with damaged (Timofeyeva et al., 2011; Timofeyeva and Fedorova, 2016; Kuznetsova et al., 2018b; Bulygin et al., 2020) or undamaged (Kuznetsova et al., 2018a, 2020) nucleotides.

**FIGURE 1:**
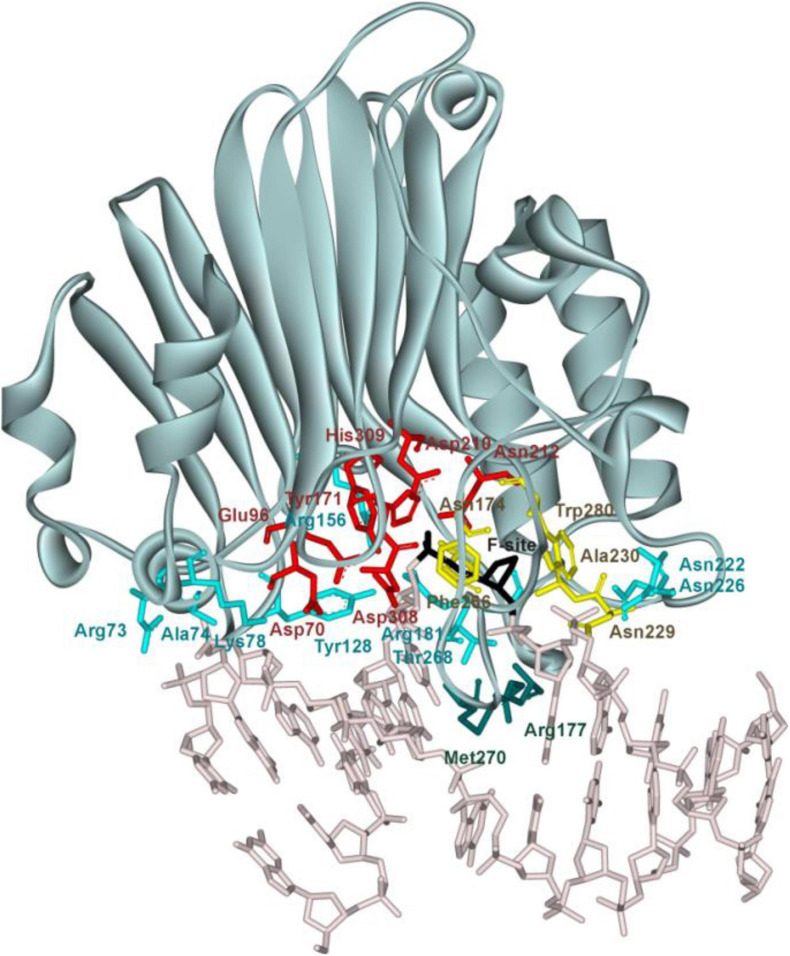
The structure of hAPE1 complexed with DNA containing an F-site (Protein Data Bank ID 1DE8). Catalytic amino acid residues Asp70, Glu96, Tyr171, Asp210, Asn212, Asp308, and His309 (red); amino acid residues of the DNA-binding site Arg73, Ala74, Lys78, Tyr128, Arg156, Arg181, Asn222, Asn226, and Thr268 (blue); intercalating amino acids Met270 and Arg177 (green); and residues Asn174, Asn229, Ala230, Phe266, and Trp280 forming a damaged base-binding pocket (yellow) are highlighted, as is the F-site (black).

Of note, at present, no three-dimensional structure of an hAPE1 complex with a DNA substrate containing a damaged base is available. Nevertheless, crystal structures of hAPE1 bound to a DNA substrate containing an F-site (an uncleavable-by-DNA-glycosylases analog of an AP-site) allow to outline amino acid network contacts that sculpt the DNA conformation in the DNA-binding site as well as to reveal the amino acid residues participating in the catalytic reaction (Figure 1; Mol et al., 2000a,b; Tsutakawa et al., 2013; Freudenthal et al., 2015). The DNA-binding site consists of Arg73, Ala74, Lys78, Tyr128, Arg156, Arg181, Asn222, Asn226, and Thr268, which preferentially form hydrogen bonds and electrostatic contacts with DNA. These contacts induce F-site eversion from the double helix into the active site of the enzyme. Two amino acid residues, Arg177 and Met270, are inserted into the DNA helix after the F-site eversion and stabilize the extrahelical state of the damage. The damaged nucleotide is placed in the pocket formed by Asn174, Asn229, Ala230, Phe266, and Trp280. Another set of amino acid residues (Asp70, Glu96, Tyr171, Asp210, Asn212, Asp308, and His309) is responsible for the coordination of the cofactor Mg^2+^ and scissile phosphate group of the damaged nucleotide. The role of Mg^2+^ ions in the DNA binding and catalytic mechanism is still debated (Gorman et al., 1997; Masuda et al., 1998; Erzberger and Wilson, 1999; Mol et al., 2000b; Beernink et al., 2001; Oezguen et al., 2007; Lipton et al., 2008; Manvilla et al., 2013; He et al., 2014; Miroshnikova et al., 2016b; Alekseeva et al., 2020).

It was demonstrated earlier (Kanazhevskaya et al., 2012) that the kinetic mechanism of the interaction between hAPE1 and an abasic DNA involves a two-step equilibrium process. Thermodynamic analysis of DNA-binding stages (Miroshnikova et al., 2016a) revealed that the formation of the primary enzyme–substrate complex includes desolvation of polar groups at the DNA–protein interface and the removal of highly ordered molecules of “crystalline water” from DNA grooves. It was assumed that at this stage, bonds are formed between the DNA and amino acid residues Arg73, Ala74, Lys78, Asn222, Asn226, Asn229, and Trp280 of the DNA-binding site. Additionally, intercalating amino acid residues Arg177 and Met270 get inserted into the DNA grooves. The second stage includes a specific rearrangement of the initial complex, e.g., flipping of the F-site into the enzyme active site and stabilization of this state by residues Arg177 and Met270 via their full insertion into the major and minor DNA grooves, respectively. Later, it was reported (Kuznetsova et al., 2018b) that after the insertion of such damaged nucleotides as εA, α-anomer of adenine (αA), DHU, and F-site into the enzyme active site, no damage-specific contacts in the binding pocket are formed between amino acid residues and the damaged DNA base. Therefore, it has been suggested that the substrate specificity of hAPE1 is governed by the ability of a damaged nucleotide to flip out from the DNA duplex in response to an enzyme-induced DNA distortion (Kuznetsova et al., 2018b). Recently, it was demonstrated by molecular dynamics simulations (Bulygin et al., 2020) that the protein loop containing Asn229/Thr233/Glu236 undergoes significant damage-dependent reorganization, indicating an important role of this loop in the recognition of a damaged nucleotide. Indeed, destabilization of the α-helix containing Thr233 and Glu236 via a loss of the interaction between these residues greatly increases the plasticity of the damaged nucleotide-binding pocket and increases the ability of the hAPE1 active site to accommodate structurally different damaged nucleotides.

Despite significant achievements in the elucidation of the mechanisms of target nucleotide recognition by AP endonucleases and in the understanding of the nature of protein–DNA contacts that control the specificity of these enzymes vis-à-vis regular and various damaged nucleotides, the question of how a particular nucleotide is recognized by the active site of the enzyme remains unanswered. Therefore, the main purpose of our study was to compare the kinetics of conformational changes of three homologous APE1-like endonucleases (insect *Drosophila melanogaster* Rrp1, amphibian *Xenopus laevis* xAPE1, and fish *Danio rerio* zAPE1) during their interaction with various damaged DNA substrates. As model damaged substrates, we used DNA duplexes containing an F-site, εA, αA, DHU, or uridine. It is noteworthy that the catalytic domains of Rrp1, xAPE1, and zAPE1 all have high amino acid sequence identity with the catalytic domain of hAPE1, thereby allowing to compare newly obtained data with the mechanism of substrate specificity control assumed earlier for hAPE1 (Kuznetsova et al., 2018b; Bulygin et al., 2020).

## Materials and Methods

### Buffers

Buffers were prepared from reagent-grade chemicals using double-distilled water. BER buffer consisting of 50 mM Tris-HCl (pH 7.5), 50 mM KCl, 5 mM MgCl_2_, 1 mM dithiothreitol, 1 mM EDTA, and 7% of glycerol (v/v). Nucleotide incision repair (NIR) buffer was composed of 50 mM Tris-HCl (pH 7.5 at 25°C), 50 mM KCl, 1 mM MgCl_2_, 1 mM dithiothreitol, 1 mM EDTA, and 7% of glycerol (v/v). All the experiments were carried out at 25°C.

### Oligonucleotides

The synthesis of the oligonucleotides (Table 1) was carried out on an ASM-800 DNA/RNA synthesizer (Biosset, Russia) by means of standard commercial phosphoramidites and CPG solid supports from the Glen Research (United States). The oligonucleotides were deprotected according to the manufacturer’s protocols and were purified by high-performance liquid chromatography. Oligonucleotide homogeneity was checked by denaturing 20% polyacrylamide gel electrophoresis (PAGE). Concentrations of oligonucleotides were calculated from their absorbance at 260 nm (A_260_). Oligonucleotide duplexes were prepared by annealing oligonucleotide strands at a 1:1 molar ratio.

**TABLE 1:** DNA substrates used in this study.

**Shorthand**	**Sequence**
F-substrate	5′-GCTCAFGTACAGAGCTG-3′ 3′-CGAGTGCATGTCTCGAC-5′
F-aPu-substrate	5′-GCTCAF(aPu)TACAGAGCTG-3′ 3′-CGAGTGTATGTCTCGAC-5′
FRET-X-substrate X = F-site, DHU or U	5′-FAM-GCTCAXGTACAGAGCTG-3′ 3′-CGAGTGCATGTCTCGAC-BHQ1-5′
FRET-Y-substrate Y = αA, εA	5′-FAM-GCTCAYGTACAGAGCTG-3′ 3′-CGAGTTCATGTCTCGAC-BHQ1-5′
Nondamaged DNA	5′-FAM-GCTCACGTACAGAGCTG-3′ 3′-CGAGTGCATGTCTCGAC-BHQ1-5′

### Enzyme Purification

hAPE1 was expressed and purified in its native form without tags or other modifications as described previously (Daviet et al., 2007). The enzymes Rrp1 (*D. melanogaster*), xAPE1 (*X. laevis*), and zAPE1 (*D. rerio*) were isolated from *Escherichia coli* Rosetta2 (DE3) cells transformed with plasmid pET28c carrying an N-terminal His_6_ and a relevant gene. To purify these enzymes expressed as recombinant proteins, 1 L of culture [in Luria–Bertani (LB) broth] of *E. coli* cells carrying the required encoding vector construct was grown with 50 μg/ml of kanamycin at 37°C until A_600_ reached 0.6–0.7; the expression of the enzymes was induced overnight with 0.3 mM isopropyl β-D-1-thiogalactopyranoside. The cells were harvested by centrifugation (5,000 × *g*, 10 min) and then resuspended in a buffer (20 mM HEPES-KOH pH 7.8, 40 mM NaCl, and 0.1% of NP40) followed by cell lysis by means of the French press. All the purification procedures were carried out at 4°C. Each homogenate was centrifuged at 40,000 × *g* for 40 min, NaCl concentration in the supernatant was brought to 250 mM (400 mM in the case of Rrp1), and the supernatant was passed through a column packed with 30 ml of Q-Sepharose Fast Flow (Cytiva, GE Healthcare Life Sciences, United States) pre-equilibrated in the same buffer. The flow-through fractions containing an enzyme were pooled, supplemented with 20 mM imidazole, and loaded on a 1-ml HiTrap-Chelating^TM^ column (Cytiva GE Healthcare Life Sciences, United States). Bound proteins were eluted with a linear 20 → 500 mM gradient of imidazole. The protein concentration was measured by the Bradford method; the stock solution was stored at −20°C.

### PAGE Experiments

6-Carboxyfluorescein (FAM)-5′-labeled oligonucleotides were subjected to experiments on separation of cleavage products by PAGE. AP endonuclease assays with all DNA substrates were carried out at 25°C in 10 μl reactions containing BER reaction buffer (in the case of the F-site-containing DNA) or NIR reaction buffer (in case of αA-, εA-, DHU-, or U-containing DNA). The substrate concentration chosen for comparing the activity of the enzymes was 1.0 μM, and the concentration of each enzyme was 1.0 μM as well. The reaction was initiated by the addition of the enzyme. Aliquots of the reaction mixture were withdrawn, immediately quenched with 10 μl of a gel-loading dye containing 7 M urea and 25 mM EDTA, followed by heating at 95°C for 3 min, and were loaded on a 20% (w/v) polyacrylamide/7 M urea gel. PAGE (gel concentration, 20%) was performed under denaturing conditions (7 M urea) at 55°C and a voltage of 200–300 V. The gels were visualized using an E-Box CX.5 TS gel-documenting system (Vilber Lourman, France), and the bands were quantified by scanning densitometry in the Gel-Pro Analyzer software, v.4.0 (Media Cybernetics, United States).

### Fluorescence Stopped-Flow Experiments

Pre-steady-state kinetics were studied by the stopped-flow technique using an SX20 stopped-flow spectrometer (Applied Photophysics Ltd., Leatherhead, United Kingdom). The fluorescence of FAM was excited at 494 nm, and the Förster resonance energy transfer (FRET) signal was monitored at wavelengths ≥530 nm by means of an OG-530 filter (Schott, Mainz, Germany). Trp was excited at 290 nm, and its fluorescence emission was monitored at wavelengths ≥320 nm using a WG-320 filter (Schott, Mainz, Germany). 2-Aminopurine (aPu) fluorescence was excited at 310 nm, and its emission was monitored at wavelengths ≥370 nm with an LG-370 Corion filter. The dead time of the instrument is 1.4 ms. Typically, each trace shown is an average of three or more individual experiments. Experimental error was less than 5%. Experiments with α-A-, ε-A-, DHU-, and U-containing DNA substrates were conducted in NIR buffer. Experiments with the F-site-containing DNA (F-substrate) and a nondamaged DNA duplex were performed in BER buffer. The solutions containing the enzyme and substrate were loaded into two separate syringes of the stopped-flow instrument and were incubated for an additional 3 min at 25°C prior to mixing. The reported concentrations of reactants are those in the reaction chamber after mixing.

### Analysis of the Kinetic Data

The sets of kinetic curves obtained at different concentrations of the F-substrate during interactions with an enzyme (zAPE1, xAPE1, or Rrp1) were fitted to the following exponential equation (Eq. 1) with amplitudes A_1_ and A_2_ and first-order rate constants *k*_obs1_ and *k*_obs2_, respectively, in the Origin software (Originlab Corp.):

(1) $$y=A_{1}\,\exp\left(-k_{o\,b\,s\,1}\,t\right)+A_{2}\,\exp\left(-k_{o\,b\,s\,2}\,t\right)+\text{offset}$$

For the linear fits of the change in observed rate constants (*k*_obs_), Eqs 2 and 3 were used:

(2) $$k_{o\,b\,s\,1}=k_{1}\,\left[\text{E}\right]+k_{-1}$$

where *k*_1_ and *k*_–__1_ are rate constants for the forward and reverse reaction, and [E] is the enzyme concentration;

(3) $$k_{o\,b\,s\,2}=k_{c\,a\,t}\,\left[\text{E}\right]$$

where *k*_cat_ is the rate constant of the irreversible catalytic step of the enzymatic reaction, and [E] denotes the concentration of the enzyme.

The sets of kinetic curves obtained at different concentrations of the non-specific DNA during interactions with an enzyme (hAPE1, zAPE1, xAPE1, or Rrp1) were analyzed in the DynaFit software (BioKin, Pullman, WA) (Kuzmic, 1996) as described elsewhere (Kuznetsov et al., 2012a,b, 2014; Kladova et al., 2018b).

The kinetic curves represent changes in the FRET signal in the course of the reaction owing to sequential formation and subsequent transformation of the DNA–enzyme complex and its conformers. The stopped-flow fluorescence traces were directly fitted to fluorescence intensity (F) at any reaction time point (t) as the sum of the intensities of background fluorescence and fluorescence of each intermediate complex formed by the enzyme with DNA:

(4) $$F=F_{b}+\sum_{i=0}^{n}f_{i}\times {\left[\text{ES}\right]}_{i}$$

where *F*_*b*_ is the background fluorescence or the equipment-related photomultiplier parameter (“noise”), and *f*_*i*_ is the molar response coefficient of the *i*-th intermediate ES*_*i*_* (*i* = 0 corresponds to the free protein and *i* > 0 to the enzyme–DNA complexes).

Concentrations of each species in the mechanisms are described by a set of differential equations according to a kinetic scheme (see section “Results”). The software performs numerical integration of a system of ordinary differential equations with subsequent nonlinear least-squares regression analysis. In the fits, the values of all relevant rate constants for the forward and reverse reactions are optimized, as are the specific molar “response factors” for all intermediate complexes.

## Results and Discussion

### The Rationale

**FIGURE 2:**
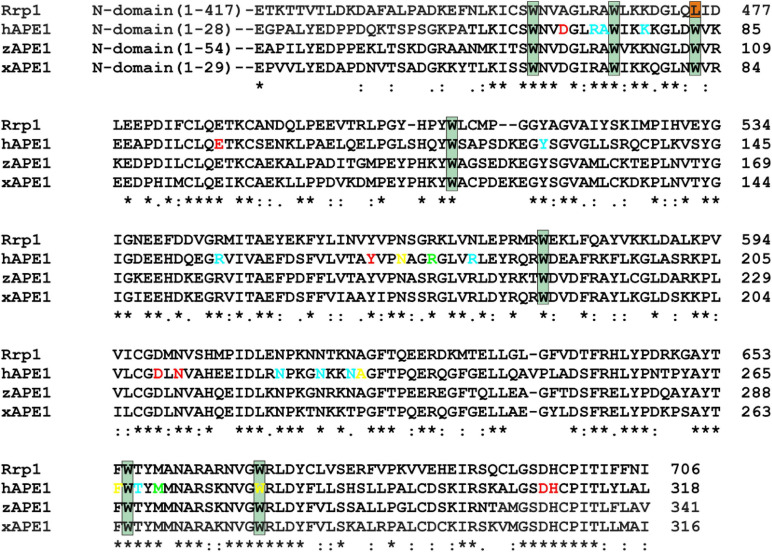
An alignment of sequences of hAPE1, xAPE1, zAPE1, and Rrp1. Catalytic amino acid residues Asp70, Glu96, Tyr171, Asp210, Asn212, Asp308, and His309 (red); amino acid residues of the DNA-binding site Arg73, Ala74, Lys78, Tyr128, Arg156, Arg181, Asn222, Asn226, Asn229, and Thr268 (blue); intercalating amino acid residues Met270 and Arg177 (light green); and residues Asn174, Asn229, Ala230, Phe266, and Trp280 forming the damaged base-binding pocket (yellow) are highlighted. Conserved Trp residues are indicated by dark green boxes. Asterisks indicate identical residues, colons denote conserved residues, and dots are residues with at least some physicochemical properties conserved.

Previously, it has been proposed that the key factor responsible for DNA substrate specificity of hAPE1 is the ability of a damaged nucleotide to get everted from a double helix and to get inserted into the damaged nucleotide-binding pocket (Kuznetsova et al., 2018b; Bulygin et al., 2020). To verify whether this mechanism is a common feature of other APE1-like enzymes, three AP endonucleases from different species were chosen on the basis of high identity of their C-terminal catalytic domain with hAPE1 (Figure 2).

Notably, the N-terminal domains of APE1-like enzymes substantially vary in size (Figure 2). Among the tested enzymes, the largest domain (consisting of 417 amino acid residues) was found in Rrp1. Moreover, the numbers of basic residues in N-terminal domains are also considerably higher in Rrp1 than in the other tested enzymes: 20 arginines and 74 lysines in Rrp1, two arginines and nine lysines in hAPE1, five arginines and nine lysines in zAPE1, and one arginine and nine lysines in xAPE1. Analysis of N-truncated variants of hAPE1 revealed that this lysine-rich region might act by stabilizing the non-specific association with nucleic acids through electrostatic interactions (Fantini et al., 2010; Poletto et al., 2013). A loss of the N-terminal domain influences the stability of both enzyme–substrate and enzyme–product complexes (Izumi and Mitra, 1998; Chattopadhyay et al., 2006) but does not affect the rates of initial complex formation or catalysis (Timofeyeva et al., 2009) in the case of abasic DNA cleavage. By contrast, this domain affects both the rate of formation and the stability of the initial complex in the cases of NIR and 3′→5′ exonuclease activities (Gros et al., 2004; Daviet et al., 2007; Timofeyeva et al., 2009). Multiple studies also underscore that the N-terminal domain may functionally interact with different proteins involved in DNA repair (Kladova et al., 2018a; Moor et al., 2020; Popov et al., 2020), transcription (Georgiadis et al., 2008; Kelley et al., 2011; Bazlekowa-Karaban et al., 2019), and RNA metabolism (Vascotto et al., 2009; Tell et al., 2010; Poletto et al., 2013). Summarizing the literature data, we can conclude that the N-terminal domain may be required for the preliminary low-affinity binding process in search of the proper lesion in DNA or a cleavage site in RNA and protein–protein interactions. Therefore, in the present study, we compare properties of APE1-like enzymes taking into account differences in the C-terminal catalytic domain, which is responsible for the specific DNA binding.

An alignment of sequences of the C-terminal catalytic domain of the four AP endonucleases revealed that almost all DNA-binding amino acid residues are identical among all these enzymes, except Arg181 (amino acid numbering corresponding to hAPE1 sequence), which is replaced by Asn in Rrp1, and Asn229 is replaced by Thr in xAPE1. Intercalating and catalytic residues are identical too, with a single substitution of Asp70, which coordinates a Mg^2+^ ion, by Ala in the case of Rrp1. It is worth noting that two amino acid residues of the damaged base-binding pocket (Asn229 and Ala230) are replaced by Thr and Pro in xAPE1.

**FIGURE 3:**
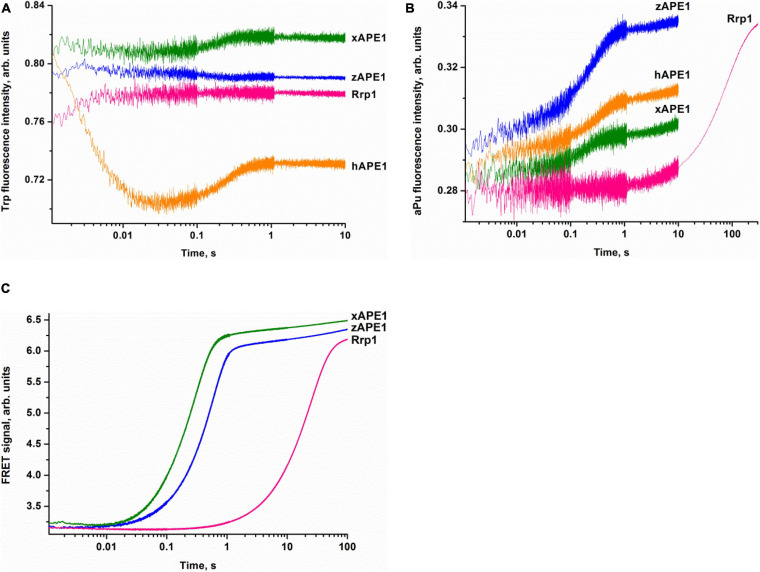
Interactions of AP endonucleases with DNA containing an F-site, as monitored by changes in the fluorescence intensity of Trp **(A)**, aPu **(B)**, or FRET signal **(C)**. (DNA substrate) = (enzyme) = 1.0 μM.

To examine the kinetics of interactions of the AP endonucleases under study with different DNA substrates, we employed the stopped-flow fluorescence method. This method is applied to the research on pre-steady-state kinetics of different enzymatic systems using various types of fluorescence (Fischer et al., 2004; Toseland and Webb, 2013; Kuznetsova et al., 2018b; Kladova et al., 2019). It is important to choose an appropriate system with which to follow the conformational changes of the DNA substrate or the enzyme. This is because the fluorescence signal change resulting from the interactions between the enzyme and substrate can be rather complex and likely will differ from one type of fluorescence to another. Accordingly, it is always useful to study the same reaction by following the changes in several types of fluorescence. Previously, a significant amount of data has been obtained on the kinetics of hAPE1 interacting with different substrates by means of the fluorescence of Trp residues in the enzyme (Timofeyeva et al., 2009, 2011; Kuznetsova et al., 2018b; Alekseeva et al., 2019b) and also DNA substrates that contain aPu as a fluorescent nucleotide analog (Kanazhevskaya et al., 2012; Kuznetsova et al., 2014, 2018a) or an emitter/quencher pair of dyes to measure FRET (Miroshnikova et al., 2016b; Alekseeva et al., 2019a, 2020). It was tempting to use the data known for hAPE1 as a reference of all APE1-like enzymes tested in the present study. Nonetheless, we were unable to detect significant changes in own Trp fluorescence intensity of xAPE1, zAPE1, and Rrp1 in the reactions with the F-substrate (Figure 3A). Indeed, the amplitude of changes in the fluorescence intensity of Trp residues during the interactions of the AP endonucleases under study with the F-substrate was extremely small or absent at all when compared with hAPE1. Of note, hAPE1 contains seven Trp residues (Trp67, Trp75, Trp83, Trp119, Trp188, Trp267, and Trp280) that are absolutely conserved in all the tested AP endonucleases (Figure 2), except for the absence of Trp83 in Rrp1. It has been suggested previously that observed changes in Trp fluorescence of hAPE1 most likely reflect conformational changes near the Trp280 residue, which is situated in the active site of hAPE1 (Miroshnikova et al., 2016a,b; Kuznetsova et al., 2018b). Even though Trp residues are conserved in all enzymes, and Trp280 is believed to make a major contribution to alterations in the intensity of the protein’s own fluorescence during the enzymatic reaction with a DNA substrate, the weak changes in the signal most likely indicate a difference in the conformational mobility of the parts of protein molecules that contain Trp residues. It can also be assumed that the difference in Trp fluorescence intensity behavior is dependent on the features of non-specific DNA binding by the N-terminal domain of enzymes. The role of the N-terminal domain in damage-specific recognition and formation of the catalytic complex remains elusive.

The pilot experiments with the F-aPu-substrate containing aPu next to the F-site (Figure 3B) revealed an increase in the fluorescence intensity of aPu within the initial 1-s period. The increase in the fluorescence intensity of aPu during DNA substrate cleavage was reported earlier for hAPE1 (Kanazhevskaya et al., 2012). Therefore, the obtained traces for xAPE1 and zAPE1 are consistent with fast cleavage of the F-site by these enzymes. In the case of Rrp1 from *D. melanogaster*, the increase phase was slower and finalized only during 300 s, implying that Rrp1 possesses a much weaker AP endonuclease activity.

To follow the conformational changes in DNA by detecting changes in the FRET signal, we used the FRET-F-duplex with FAM attached to the 5′ end of the damaged strand and a BHQ1 quencher residue attached to the 5′ end of the complementary strand. It should be noted that FRET detection has been previously utilized for the analysis of DNA cleavage activity of hAPE1 (Alekseeva et al., 2019a, 2020; Kladova et al., 2020). As shown in Figure 3C, FRET detection revealed a significant increase in the signal that corresponds to catalytic cleavage of the F-site for all the tested AP endonucleases. As in the previous case, F-site cleavage by Rrp1 was notably slower than that by other AP endonucleases.

Overall, the comparative analysis of kinetic traces, which reflect (i) potential conformational variation of the enzyme molecule after catalytic activity, (ii) environment changes for a single aPu residue in the substrate, and (iii) the distance between terminal fluorophores in the duplex substrate, revealed a difference among the enzymes from the insect *D. melanogaster*, amphibian *X. laevis*, and fish *D. rerio* and well-studied human APE1. Despite high identity of the functionally characterized amino acid residues among all the enzymes, it was found that Rrp1 has a considerably lower AP endonuclease activity. There are several possible reasons for the slower Rrp1 activity in comparison with xAPE1 and zAPE1. First of all, analysis of the amino acid residues in the DNA-binding site revealed that Arg181 (conserved among the tested APE1-like enzymes, Figure 2) is substituted by Asn in the case of Rrp1. On the other hand, Rrp1 features a substitution of the conserved Asp70 residue by Ala. Finally, the difference in Trp fluorescence intensity changes—between the proteins (hAPE1 and the tested enzymes) with identical spatially located Trp residues in the catalytic domain—suggests that the N-terminal domain may affect conformational mobility of the full-length enzyme. Indeed, even though the role of the N-terminal domain is unclear, it should be noted that the slowest enzyme Rrp1 has the largest N-terminal domain among the tested enzymes.

### Interaction With a DNA Oligo Containing an F-Site

**FIGURE 4:**
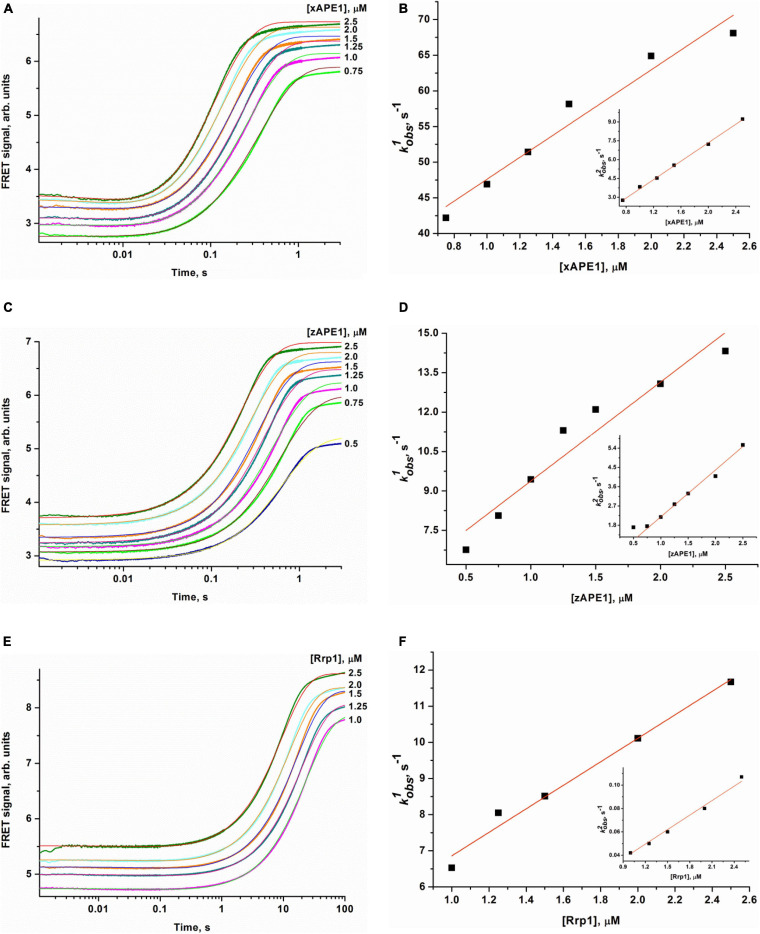
Changes in the FRET signal during the interaction of xAPE1 **(A,B)**, zAPE1 **(C,D)**, or Rrp1 **(E,F)** with the FRET-F-substrate. (FRET-F) = 1.0 μM, and concentrations of enzymes are shown on the right in the panel. Individual traces were fitted to double-exponential Eq. 1, and the dependences of the observed rate constants *k*_obs1_ and *k*_obs2_ on enzyme concentration were linearly fitted to Eqs 2 and 3, respectively.

To determine the kinetics of interactions between the DNA substrate containing an F-site (a stable synthetic analog of an AP-site) and an AP endonuclease (zAPE1, xAPE1, or Rrp1), a fixed concentration of the FRET-F-substrate was rapidly mixed with various concentrations of an enzyme by the stopped-flow apparatus, and fluorescence was recorded for some time (Figure 4). All three enzymes yielded similar patterns of the kinetic traces: a short lag was followed by a fast increase (in the FRET signal owing to the growing distance between FAM and BHQ1 residues) corresponding to the catalytic stage of the enzymatic reaction, and then all fluorescence curves reached a plateau. It must be noted that the FRET curves in case of xAPE1 featured a small but real instant decrease in the signal in the initial part of curves up to 10–20 ms (Figure 4A). This decrease phase most likely reflects the formation of the enzyme–substrate complex. Nevertheless, a substantial amplitude of the increase in the signal (reflecting the process of F-site cleavage) made it difficult to detect changes in the FRET signal (much weaker in amplitude) related to processes of substrate–enzyme complex formation in the cases of zAPE1 and Rrp1 (Figures 4C,E). This observation also implies that xAPE1 induces greater bending of the DNA substrate during the complex formation, and thus the convergence of FAM and BHQ1 residues makes the decrease in the FRET signal more detectable.

**Scheme 1:**
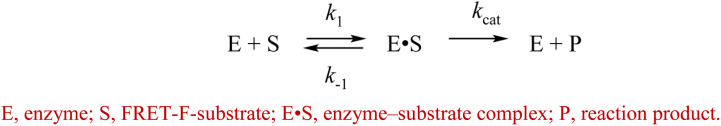
Recognition of the FRET-F-substrate by AP endonucleases.

The enzymatic cleavage of the FRET-F-substrate was generally completed by 1.0 s in the case of zAPE1 and xAPE1 and continued up to 100 s in the case of Rrp1. The traces were well fitted to a double exponential Eq. 1 giving both observed rate constants being linearly dependent on the enzyme concentration. This fitting yielded the kinetic model depicted in Scheme 1. This kinetic model is consistent with the time courses in Figure 4 and contains one equilibrium stage of the substrate binding and an irreversible catalytic stage. All rate and equilibrium constants corresponding to this mechanism are given in Table 2. Although the rate constants obtained for the enzyme–substrate complex formation gave rather similar binding constants *K*_1_ for the three enzymes, there were significant differences among their catalytic rate constants. The most rapid *k*_*cat*_ was observed for the xAPE1 enzyme (3.7 ± 0.1 s^–1^), and this constant was only slightly faster than that obtained for zAPE1 (2.2 ± 0.1 s^–1^). Rate constant *k*_cat_ characterizing catalytic cleavage of the F-site by Rrp1 turned out to be two orders of magnitude slower: 0.040 ± 0.001 s^–1^. Thus, despite the finding that the overall FRET-F-substrate-binding process takes place with similar efficacy for zAPE1, xAPE1, and Rrp1, the catalytic stage of the cleavage reaction is dramatically slowed down in the interactions with Rrp1. Probably, this loss of catalytic efficiency is associated with the lack of the Asp70 residue in the active site of Rrp1; this residue is essential for proper coordination of Mg^2+^. This assumption is in agreement with the reported moderate reduction in hAPE1 AP endonuclease activity after site-directed mutation D70A is introduced (Erzberger and Wilson, 1999).

**TABLE 2:** Rate and equilibrium constants of the interaction of AP endonucleases with the FRET-F-substrate.

**Constant**	**zAPE1**	**xAPE1**	**Rrp1**
*k*_1_× 10^−6^, M^−1^s^−1^	3.8 ± 0.4	15 ± 2	3.3 ± 0.2
*k*_–1_, s^–1^	5.6 ± 0.7	32 ± 3	3.6 ± 0.4
*K*_1_× 10^−6^, M^−1^	0.7 ± 0.2	0.5 ± 0.1	0.9 ± 0.2
*k*_*cat*_, s^–1^	2.2 ± 0.1	3.7 ± 0.1	0.040 ± 0.001

Data are presented as mean ± SD. * K_1_ = k_1_/k _– 1._

Our data allow us to conclude that the difference in the N-terminal domain among the enzymes has no effect on the catalytic complex formation with F-site-containing DNA, in good agreement with the results obtained previously for the wild type and an N-terminally truncated version of hAPE1 (Izumi and Mitra, 1998; Chattopadhyay et al., 2006; Timofeyeva et al., 2009). Moreover, the similarity of binding constants (Table 2) among all these enzymes indicates that the R181N substitution in the DNA-binding site of Rrp1 is also not essential for the catalytic complex formation with F-site-containing DNA. These findings are consistent with one study (Freudenthal et al., 2015), which revealed that wild-type and R181A hAPE1 bind to the substrate with similar affinity. Nonetheless, their binding analysis with product DNA indicates that the mutant (R181A) enzyme binds with approximately threefold weaker affinity than that of the wild-type enzyme, implying that Arg181 participates in product DNA binding.

### Binding of Nondamaged DNA

**FIGURE 5:**
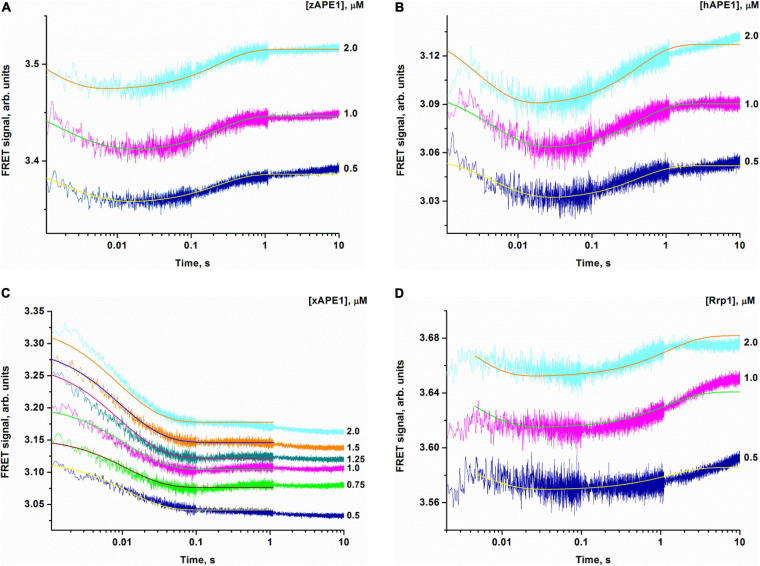
Changes in the FRET signal during the interaction of zAPE1 **(A)**, hAPE1 **(B)**, xAPE1 **(C)**, or Rrp1 **(D)** with the nondamaged DNA. (DNA) = 1.0 μM, and concentrations of enzymes are shown on the right in the panel. Traces were fitted by nonlinear least-squares regression analysis.

Crystal structures have shown that in the catalytic complex, hAPE1 induces DNA bending by approximately 35° (Mol et al., 2000a,b; Tsutakawa et al., 2013; Freudenthal et al., 2015). Damaged DNA bending has also been detected in solution by PELDOR spectroscopy and FRET analysis (Kuznetsova et al., 2018b; Alekseeva et al., 2019a). Therefore, to resolve the nondamaged DNA-binding stage, we recorded time-dependent changes of the FRET signal during DNA binding under the same conditions as those used for the FRET-F-substrate. For the nondamaged DNA, changes in the FRET signal were associated with changes in the distance between the emitter and quencher, which are located at the opposite ends of the duplex. The FRET pair of dyes could be spatially close during “endonuclease-type” complex formation in the same manner as with the damaged duplex that would lead to DNA bending. Another possible reason for the FRET signal change is the end of the binding process resulting in the formation of a “3′-5′ exonuclease-type” enzyme–DNA complex, which causes displacement of the dyes on both sides of the duplex. On the other hand, the 3′-5′ exonuclease degradation of the duplex was not observed in the selected time range (10 s, Figure 5), in line with the very slow processing rate of the blunt-end duplex compared with recessed DNA (Kuznetsova et al., 2018a; Liu et al., 2021).

When a fixed concentration of the intact DNA duplex was mixed with various concentrations of the enzymes, an initial decrease in the FRET signal was observed that was followed by an increase in the signal for zAPE1, hAPE1, and Rrp1 but not for xAPE1 (Figure 5). The experiments with well-studied hAPE1 have been conducted to obtain the reference data under the conditions closest to those used for the enzymes being tested. In the assay of xAPE1, only a decrease in the FRET signal was detectable; it manifested the biggest amplitude change among all the enzymes, suggesting that the DNA binding by xAPE1 leads to more pronounced bending of the DNA structure.

Inspection of the time courses obtained for nondamaged-DNA binding by the AP endonucleases zAPE1, xAPE1, and hAPE1 revealed that the process was essentially completed within 1 s. The time courses of interactions between intact DNA and Rrp1 reach a plateau at time points exceeding 10 s, meaning that the process of DNA binding by Rrp1 proceeded more slowly than that of the other APE1-like enzymes.

**Scheme 2:**
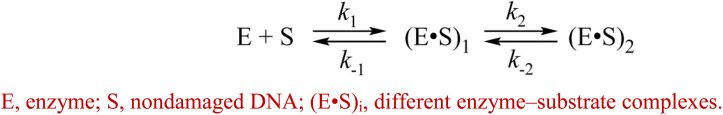
Binding of the nondamaged DNA by AP endonucleases hAPE1, zAPE1, and Rrp1.

**Scheme 3:**
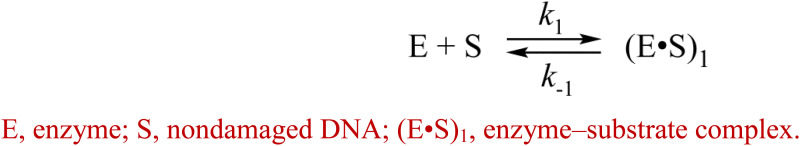
Recognition of the intact DNA by xAPE1.

The kinetic curves (Figure 5) were fitted in DynaFit to calculate rate constants of the DNA-binding steps. The analysis yielded a minimal kinetic mechanism containing two reversible steps in the case of enzymes hAPE1, zAPE1, and Rrp1 (Scheme 2) but only one-step binding for xAPE1 (Scheme 3). Rate constants of forward and reverse reactions in Schemes 2, 3 as well as the equilibrium constants calculated as the ratio of *k*_*i*_ to *k*_– *i*_ (*K*_*i*_ = *k*_*i*_/*k*_– *i*_) and the overall association constants *K*_*ass*_ = *K*_1_ *× K*_2_ (if there are any) are presented in Table 3.

**TABLE 3:** Rate and equilibrium constants of the interaction of AP endonucleases with the nondamaged DNA as determined by nonlinear least-squares regression analysis of the FRET traces.

**Constant**	**zAPE1**	**xAPE1**	**Rrp1**	**hAPE1**
*k*_1_× 10^−6^, M^−1^s^−1^	79060	705	12030	18020
*k*_–1_, s^–1^	7.00.8	5.02.0	6020	307
*K*_1_× 10^−6^, M^−1^	11020	147	2.01.0	6.02.0
*k*_2_, s^–1^	0.180.03	–	0.020.01	0.080.01
*k*_–2_, s^–1^	4.10.2	–	0.80.1	2.30.1
*K*_2_	0.040.01	–	0.030.01	0.040.01
*K*_ass_× 10^−6^, M^−1^	5.02.0	–	0.060.05	0.20.1

* K_*i*_ = k_*i*_/k _–__i_, K_*ass*_ = K_1_ × K_2._

The formation of the primary enzyme–substrate complex (E•S)_1_ was most effective for zAPE1 because forward-reaction constant *k*_1_ was the fastest among the enzymes under study (790 × 10^6^ M^–1^s^–1^) and because reverse reaction constant *k*_– 1_ [characterizing the stability of the (E•S)_1_ complex] was relatively slow (7.0 s^–1^). Another enzyme showing approximately the same low *k*_– 1_ value (5.0 s^–1^) was xAPE1. Nonetheless, for xAPE1, forward reaction constant *k*_1_ (70 × 10^6^ M^–1^s^–1^) was one order of magnitude lower compared with zAPE1, thus making association constant *K*_1_ 14 × 10^6^ M^–1^ for xAPE1, while *K*_1_ for zAPE1 was almost eifgtfold higher (110 × 10^6^ M^–1^). Forward reaction constants *k*_1_ for Rrp1 and hAPE1 were quite similar (120 × 10^6^ and 180 × 10^6^ M^–1^s^–1^, respectively) giving the lowest values of initial DNA-binding constant *K*_1_ 2.0 × 10^6^ and 6.0 × 10^6^ M^–1^, respectively.

Thus, the (E•S)_1_ complex resulting from the binding of the nondamaged DNA by the zAPE1 enzyme turned out to be formed much more efficiently and was the most stable, with its association constant *K*_1_ being an order of magnitude higher when compared with the other enzymes. Association constants *K*_1_ are similar between hAPE1 and xAPE1, but *K*_1_ for Rrp1 is the smallest one. These differences are seen in FRET signal time courses of these enzymes. The phase of the initial decrease in the FRET signal ended approximately within 0.02, 0.04, and 0.1 s, respectively, for zAPE1, hAPE1, and Rrp1. Even though the phase of fluorescence declines in the case of xAPE1 finished rather late (compared with the other enzymes), this phenomenon is associated with the absence of a pronounced phase of a subsequent increase in the FRET signal characterizing the transformation of the (E•S)_1_ complex to the (E•S)_2_ complex. Indeed, the nature of complex (E•S)_2_ requires additional elucidation because the formation of this complex is not a universal feature of the tested APE1-like enzymes. Probably, in the interaction with nondamaged DNA, this complex characterizes the efficiency of enzymes’ binding to the 3′ end of the duplex in an attempt to start 3′-5′-exonuclease degradation of the DNA.

Of note, although the forward and reverse constants [characterizing the transformation of the enzyme–substrate complex (E•S)_1_ into (E•S)_2_] showed almost ninefold differences from the *k*_2_ constant, *K*_2_ was roughly similar among all the enzymes except for xAPE1. As a result, differences in total association constant *K*_*ass*_ are completely explained by the differences in association constant *K*_1_ (characterizing the efficiency of the initial DNA binding). Overall, it can be concluded that for Rrp1, the formation of the (E•S)_2_ enzyme–substrate complex was the least efficient among the tested enzymes; this finding may be related to a possible effect of the largest N-terminal domain or of substitution R181N in the DNA-binding site of Rrp1. Therefore, these features of Rrp1 could play some role in the primary-complex transformation during the interaction with the nondamaged DNA. Nevertheless, in the case of binding of the DNA containing the F-site (Table 2), the efficiency of catalytic-complex formation was quite similar among all the APE1-like enzymes, suggesting that the efficiency of formation of different complexes and their conformational transformation are dependent on the nature of the DNA bound by these enzymes.

### Interaction With DNA Containing a Damaged Base

**FIGURE 6:**
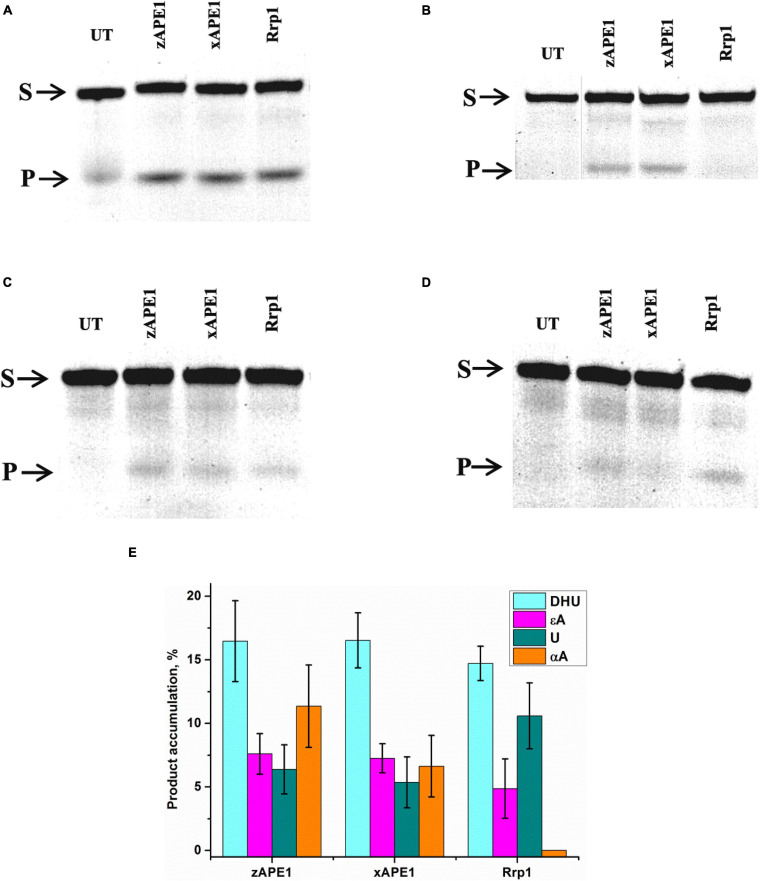
PAGE analysis of DNA cleavage by APE1-like enzymes during an interaction with the FRET–DHU-substrate **(A)**, FRET–αA-substrate **(B)**, FRET–εA-substrate **(C)**, or FRET-U-substrate **(D)**. A comparison of efficacy of model DNA substrates’ cleavage by APE1-like enzymes **(E)**. (Enzyme) = (DNA substrate) = 1.0 μM. T = 25°C, reaction time = 2 h. S is a substrate, P is a product of DNA chain cleavage, and UT is a sample untreated with an enzyme.

First of all, the cleavage efficiency of DNA substrates containing αA, εA, DHU, or uracil (U) by the three nonhuman AP endonucleases was analyzed by PAGE (Figures 6A–D). As shown in Figure 6E, all APE1-like enzymes are able to cleave DNA substrates containing DHU, εA, or U. Unexpectedly, it was found that Rrp1 is inactive toward αA-containing DNA, whereas xAPE1 and zAPE1 can still recognize this lesion as a substrate. Regarding the activity of hAPE1 toward various target nucleotides, it has been reported (Prorok et al., 2013; Kuznetsova et al., 2018b; Bulygin et al., 2020) that αA is a better substrate than εA and U. These findings indicate that despite the high identity of active-site residues among all the tested APE1-like enzymes, they have individual features of substrate recognition.

**FIGURE 7:**
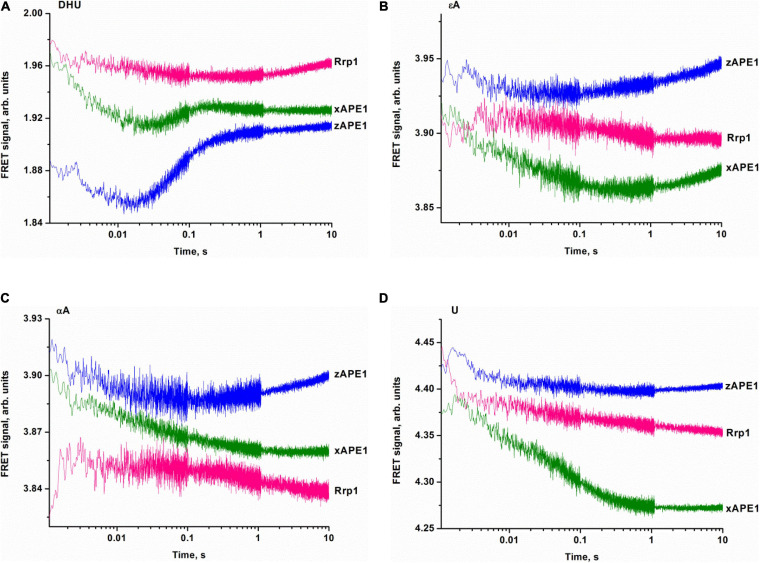
Binding of APE1-like enzymes with the FRET–DHU-substrate **(A)**, FRET–εA-substrate **(B)**, FRET–αA-substrate **(C)**, or FRET–U-substrate **(D)**. (Enzyme) = (DNA substrate) = 1.0 μM.

To clarify DNA conformational changes in the course of substrate binding, fluorescence curves reflecting the interactions of AP endonucleases with different DNA substrates (containing αA, εA, DHU, or U) were recorded by the stopped-flow kinetic method (Figure 7). A very low rate of site-specific DNA cleavage together with interference with 3′–5′ exonuclease degradation of DNA substrates under some conditions with long reaction time did not let us register the full enzyme cycle, which includes DNA binding, cleavage, and product dissociation. Therefore, the experimental limitations did not allow us to identify a kinetic model for damaged DNA substrates. All time courses are presented in time intervals up to 10 s corresponding only to DNA-binding steps because—as was demonstrated by PAGE—no significant cleavage of these DNA substrates occurs in the given time interval. Thus, all changes in the FRET signal depicted in Figure 7 characterize only the initial binding of the DNA substrate by the enzymes and subsequent conformational transformation of the enzyme–substrate complexes. The complexity of the obtained time courses and a low signal-to-noise ratio in some cases did not permit precise fitting of the kinetic curves.

The fluorescence time courses recorded for interactions between the AP endonucleases and DHU-containing DNA substrate revealed a decrease in the FRET signal followed by a subsequent increase as in the case of nondamaged DNA (Figure 7A). The most pronounced changes in the FRET signal were observed when the DHU-substrate was bound by zAPE1 and xAPE1. The fast initial decrease in the FRET signal that proceeds within 0.02 s most likely reflects the emergence of the primary enzyme–substrate complex. This phase for Rrp1 was at least 10-fold slower and finalized only at time point “0.3 s.” Because of the slow rate of primary-complex formation, the second phase of the FRET signal increase was also significantly slower for Rrp1 when compared with xAPE1 and zAPE1.

Fluorescence time courses obtained for the interactions of zAPE1 and xAPE1 with the εA-containing DNA substrate (Figure 7B) showed an initial decrease in the FRET signal up to time point 0.02 and 0.4 s, respectively, followed by an increase in the signal. For the Rrp1 interaction with the FRET-εA-substrate, only a decrease phase of up to the 10 s was detectable. These data revealed that the larger εA base than DHU base is recognized by APE1-like enzymes with completely different efficiency. In the assessment of εA-DNA recognition by Rrp1, the initial binding step was expectedly slower compared with the other enzymes, in agreement with the data on the nondamaged DNA. Nevertheless, the 20-fold rate difference between zAPE1 and xAPE1 revealed a significant disturbance of εA base recognition in comparison with the DHU base (Figures 7A,B). As recently reported regarding hAPE1 (Bulygin et al., 2020), accommodation of a damaged base in the active-site pocket is associated with a significant disturbance of the “damage recognition loop,” which includes residues Asn229, Thr233, and Glu236. An alignment of amino acid sequences of the tested enzymes (Figure 2) revealed that Thr233 and Glu236 are fully conserved, whereas Asn229 is substituted by Thr in xAPE1. It is also worth noting that the human APE1 Asn229 mutant shows significantly weaker binding to a damaged DNA substrate (Shen and Loeb, 2003; Izumi et al., 2004). Moreover, Ala230—forming the wall of the damaged base-binding pocket—is also substituted by the rigid Pro residue only in xAPE1. Therefore, when interacting with an abasic nucleotide or even DHU-containing DNA, highly similar zAPE1 and xAPE1 significantly differ in the rate of formation of the initial complex and its subsequent transformation during an interaction with a larger damaged nucleotide containing the εA base. Moreover, the data obtained about the αA- and U-containing DNA substrates (Figures 7C,D) uncovered slower initial complex formation for xAPE1 in comparison with zAPE1. Indeed, on the one hand, molecular dynamics simulations (Bulygin et al., 2020) indicate that during the recognition of various damaged nucleotides, the DNA-binding site of APE1-like enzymes must undergo conformational changes to accommodate the nucleotide containing a damaged base. On the other hand, as reported (Bulygin et al., 2020), the cleavage of αA- and εA-substrates is more or less efficient because the position of these bases in the binding pocket does not correspond to optimal distances between the scissile phosphate group and catalytic amino acid residues.

The detailed molecular mechanism underlying the control of specificity to a damaged base requires additional investigation including site-directed mutational analysis of some enzymes and global evolutionary analysis of these enzymes to identify possible roles of amino acid residues of the enzyme in the mechanism of substrate recognition. Moreover, the impact of the N-terminal domain on the DNA-binding properties and ability of enzymes to recognize different types of damaged bases should be researched further. Nevertheless, the obtained data support the idea that the NIR activity of AP endonucleases is a generic function of these enzymes.

## Conclusion

In general, our examination of fluorescence time courses—obtained for the interaction of several APE1-like enzymes with DNA substrates containing various lesions and a FRET dye pair—allows us to outline a model of substrate recognition by this class of enzymes. Binding constants for F-site-containing DNA proved to be similar among all the assayed AP endonucleases, suggesting that abasic site recognition by these enzymes most likely is based on a combination of three “whales”: DNA flexibility, the ability of the F-site to get flipped out from the duplex, and the absence of steric hindrances throughout the trajectory of eversion into the active site of the enzymes. However, Rrp1 manifested a significantly slower rate of the catalytic reaction with F-site-containing DNA compared with zAPE1 and xAPE1. This effect is possibly related to the loss of Asp70, one of the residues that coordinate cofactor Mg^2+^ in the active site. This assumption is in agreement with the moderately lower AP endonuclease activity of hAPE1 carrying site-directed mutation D70A (Erzberger and Wilson, 1999).

The binding processes leading to the changes in the FRET signal during interactions with nondamaged DNA and DNA substrates containing damaged bases are the fastest for zAPE1, and the rates of these processes are comparable to those of hAPE1 reported previously (Kuznetsova et al., 2018b; Bulygin et al., 2020). Of note, the substitution of two amino acid residues in the damaged base-binding pocket (Asn229Thr and Ala230Pro) in xAPE1 in comparison with the other APE1-like enzymes (hAPE1, zAPE1, and Rrp1) leads to significant differences in the rates of formation of the initial complex with DNA containing αA, εA, or U but not the less rigid F-site or the nonplanar DHU base. For Rrp1, the binding of nondamaged DNA and DNA substrates containing damaged bases was the slowest. Even though the substitution of Arg181 in the DNA-binding site by the Asn residue does not affect catalytic complex formation in the case of F-site-containing DNA, this mutation may alter the non-specific binding and recognition of bulkier nucleotides containing a damaged base. Another possible factor that could influence the DNA-binding process is the N-terminal domain of the enzymes under study. Although the role of N-terminal domains in the DNA binding and recognition of a damaged base is outside the scope of this study, it is likely that the lower DNA-binding affinity for nondamaged DNA and DNA substrates containing damaged bases is linked with differences in this domain. As suggested for hAPE1, the N-terminal domain influences the rate of formation of the enzyme–substrate complex in NIR and 3′→5′ exonuclease reactions (Gros et al., 2004; Daviet et al., 2007; Timofeyeva et al., 2009) but not in the case of abasic-DNA cleavage (Izumi and Mitra, 1998; Chattopadhyay et al., 2006).

Unexpectedly, the differences in the rates of DNA substrates’ binding do not cause significant differences in the cleavage efficiency of the DNA containing a damaged base, suggesting that the formation of enzyme–substrate complexes is not the key factor that limits the enzyme turnover. From this standpoint, individual site-directed mutations in the active site or the whole N-domain are not important for the ability of the enzyme to recognize a damaged base; they can only affect the rate of complex formation. Our results suggest that the nature of damage recognition and cleavage efficacy have something to do with the fine conformational tuning inside the active site. In this regard, our results on APE1-like enzymes are in agreement with our findings about hAPE1 (Bulygin et al., 2020): the location of αA and εA in the binding pocket does not correlate with the conformation of amino acid residues optimal for catalysis. Therefore, the conformational rearrangements inside the active site must be the driving force behind the processes catalyzed by APE1-like enzymes.

Taken together, our results mean that the activity of AP endonucleases toward a damaged base-containing DNA is a common feature of AP endonucleases from evolutionarily distant species, as initially reported for Nfo from *E. coli* (Ide et al., 1994; Ischenko and Saparbaev, 2002; Ishchenko et al., 2004) and later described for hAPE1 (Gros et al., 2004). It is well known that hAPE1 is an essential enzyme for abasic-site cleavage in the BER pathway and in transcriptional regulation of genes. Moreover, a knockout of the *APE1* gene in mice or even a knockdown of APE1 activity increases the sensitivity to oxidative stress and promotes cell death (Xanthoudakis, 1996; Meira et al., 2001; Huamani et al., 2004; Fung and Demple, 2005; Unnikrishnan et al., 2009) implying importance of this enzyme. On the other hand, at present, it is still unclear whether inactivation of which function of this multifunctional enzyme has such serious consequences. Therefore, our data suggest that the NIR activity of AP endonucleases is conserved among insects (*D. melanogaster*), amphibians (*X. laevis*), fishes (*D. rerio*), and humans, indicating its high importance for cellular processes.

## Data Availability Statement

The original contributions presented in the study are included in the article/supplementary material, further inquiries can be directed to the corresponding authors.

## Author Contributions

ATD conducted the experiments. AAI, MS, and NAK conceived and designed the experiments. AID, NAK, and OSF analyzed the data. AAI, MS, NAK, and OSF contributed to the reagents, materials, and/or analytical tools. ATD, NAK, MS, and OSF wrote the manuscript. All authors contributed to the article and approved the submitted version.

## Conflict of Interest

The authors declare that the research was conducted in the absence of any commercial or financial relationships that could be construed as a potential conflict of interest.

## References

[B1] Alekseeva I. V. Bakman A. S. Vorobjev Y. N. Fedorova O. S. Kuznetsov N. A. (2019a). Role of ionizing amino acid residues in the process of DNA binding by human AP endonuclease 1 and in its catalysis. *J. Phys. Chem. B* 123 9546–9556. 10.1021/acs.jpcb.9b07150 31633353

[B2] Alekseeva I. V. Davletgildeeva A. T. Arkova O. V. Kuznetsov N. A. Fedorova O. S. (2019b). The impact of single-nucleotide polymorphisms of human apurinic/apyrimidinic endonuclease 1 on specific DNA binding and catalysis. *Biochimie* 163 73–83. 10.1016/j.biochi.2019.05.015 31150756

[B3] Alekseeva I. V. Kuznetsova A. A. Bakman A. S. Fedorova O. S. Kuznetsov N. A. (2020). The role of active-site amino acid residues in the cleavage of DNA and RNA substrates by human apurinic/apyrimidinic endonuclease APE1. *Biochim. Biophys. Acta Gen. Subj.* 1864 129718. 10.1016/j.bbagen.2020.129718 32858086

[B4] Barnes T. Kim W. C. Mantha A. K. Kim S. E. Izumi T. Mitra S. (2009). Identification of Apurinic/apyrimidinic endonuclease 1 (APE1) as the endoribonuclease that cleaves c-myc mRNA. *Nucleic Acids Res.* 37 3946–3958. 10.1093/nar/gkp275 19401441 PMC2709568

[B5] Barzilay G. Hickson I. D. (1995). Structure and function of apurinic/apyrimidinic endonucleases. *Bioessays* 17 713–719. 10.1002/bies.950170808 7661852

[B6] Bazlekowa-Karaban M. Prorok P. Baconnais S. Taipakova S. Akishev Z. Zembrzuska D. (2019). Mechanism of stimulation of DNA binding of the transcription factors by human apurinic/apyrimidinic endonuclease 1, APE1. *DNA Repair (Amst.)* 82:102698. 10.1016/j.dnarep.2019.102698 31518879

[B7] Beernink P. T. Segelke B. W. Hadi M. Z. Erzberger J. P. Wilson D. M. III Rupp B. (2001). Two divalent metal ions in the active site of a new crystal form of human apurinic/apyrimidinic endonuclease, Ape1: implications for the catalytic mechanism. *J. Mol. Biol.* 307 1023–1034. 10.1006/jmbi.2001.4529 11286553

[B8] Berquist B. R. McNeill D. R. Wilson D. M. III (2008). Characterization of abasic endonuclease activity of human Ape1 on alternative substrates, as well as effects of ATP and sequence context on AP site incision. *J. Mol. Biol.* 379 17–27. 10.1016/j.jmb.2008.03.053 18439621 PMC2430724

[B9] Bulygin A. A. Kuznetsova A. A. Vorobjev Y. N. Fedorova O. S. Kuznetsov N. A. (2020). The role of active-site plasticity in damaged-nucleotide recognition by human apurinic/apyrimidinic endonuclease APE1. *Molecules* 25:3940. 10.3390/molecules25173940 32872297 PMC7504742

[B10] Chattopadhyay R. Wiederhold L. Szczesny B. Boldogh I. Hazra T. K. Izumi T. (2006). Identification and characterization of mitochondrial abasic (AP)-endonuclease in mammalian cells. *Nucleic Acids Res.* 34 2067–2076. 10.1093/nar/gkl177 16617147 PMC1440881

[B11] Chou K.-M. Cheng Y.-C. (2003). The exonuclease activity of human apurinic/apyrimidinic endonuclease (APE1). Biochemical properties and inhibition by the natural dinucleotide Gp4G. *J. Biol. Chem.* 278 18289–18296. 10.1074/jbc.M212143200 12624104

[B12] Daviet S. Couve-Privat S. Gros L. Shinozuka K. Ide H. Saparbaev M. (2007). Major oxidative products of cytosine are substrates for the nucleotide incision repair pathway. *DNA Repair* 6 8–18. 10.1016/j.dnarep.2006.08.001 16978929

[B13] Demple B. Sung J.-S. (2005). Molecular and biological roles of Ape1 protein in mammalian base excision repair. *DNA Repair* 4 1442–1449. 16199212 10.1016/j.dnarep.2005.09.004

[B14] Erzberger J. P. Wilson D. M. III (1999). The role of Mg2+ and specific amino acid residues in the catalytic reaction of the major human abasic endonuclease: new insights from EDTA-resistant incision of acyclic abasic site analogs and site-directed mutagenesis. *J. Mol. Biol.* 290 447–457. 10.1006/jmbi.1999.2888 10390343

[B15] Fantini D. Vascotto C. Marasco D. D’Ambrosio C. Romanello M. Vitagliano L. (2010). Critical lysine residues within the overlooked N-terminal domain of human APE1 regulate its biological functions. *Nucleic Acids Res.* 38 8239–8256. 10.1093/nar/gkq691 20699270 PMC3001066

[B16] Fischer C. J. Maluf N. K. Lohman T. M. (2004). Mechanism of ATP-dependent translocation of E. coli UvrD monomers along single-stranded DNA. *J. Mol. Biol.* 344 1287–1309. 10.1016/j.jmb.2004.10.005 15561144

[B17] Freudenthal B. D. Beard W. A. Cuneo M. J. Dyrkheeva N. S. Wilson S. H. (2015). Capturing snapshots of APE1 processing DNA damage. *Nat. Struct. Mol. Biol.* 22 924–931. 10.1038/nsmb.3105 26458045 PMC4654669

[B18] Friedberg E. C. Roger A. S. Wolfram S. Graham C. W. Tom E. Richard D. W. (2006). *DNA Repair and Mutagenesis, Second Edition.* Washington, DC: American Society of Microbiology, 10.1128/9781555816704

[B19] Fromme J. C. Banerjee A. Verdine G. L. (2004). DNA glycosylase recognition and catalysis. *Curr. Opin. Struct. Biol.* 14 43–49. 15102448 10.1016/j.sbi.2004.01.003

[B20] Fung H. Demple B. (2005). A vital role for Ape1/Ref1 protein in repairing spontaneous DNA damage in human cells. *Mol. Cell* 17 463–470. 10.1016/j.molcel.2004.12.029 15694346

[B21] Georgiadis M. M. Luo M. Gaur R. K. Delaplane S. Li X. Kelley M. R. (2008). Evolution of the redox function in mammalian apurinic/apyrimidinic endonuclease. *Mutat. Res.* 643 54–63. 10.1016/j.mrfmmm.2008.04.008 18579163 PMC2637456

[B22] Gorman M. A. Morera S. Rothwell D. G. de La Fortelle E. Mol C. D. Tainer J. A. (1997). The crystal structure of the human DNA repair endonuclease HAP1 suggests the recognition of extra-helical deoxyribose at DNA abasic sites. *EMBO J.* 16 6548–6558. 10.1093/emboj/16.21.6548 9351835 PMC1170259

[B23] Gros L. Ishchenko A. A. Ide H. Elder R. H. Saparbaev M. K. (2004). The major human AP endonuclease (Ape1) is involved in the nucleotide incision repair pathway. *Nucleic Acids Res.* 32 73–81. 10.1093/nar/gkh165 14704345 PMC373275

[B24] Gros L. Saparbaev M. K. Laval J. (2002). Enzymology of the repair of free radicals-induced DNA damage. *Oncogene* 21 8905–8925. 12483508 10.1038/sj.onc.1206005

[B25] He H. Chen Q. Georgiadis M. M. (2014). High-resolution crystal structures reveal plasticity in the metal binding site of apurinic/apyrimidinic endonuclease I. *Biochemistry* 53 6520–6529. 10.1021/bi500676p 25251148 PMC4204877

[B26] Huamani J. McMahan C. A. Herbert D. C. Reddick R. McCarrey J. R. MacInnes M. I. (2004). Spontaneous mutagenesis is enhanced in apex heterozygous mice. *Mol. Cell. Biol.* 24 8145–8153. 10.1128/MCB.24.18.8145-8153.2004 15340075 PMC515041

[B27] Ide H. Tedzuka K. Shimzu H. Kimura Y. Purmal A. A. Wallace S. S. (1994). α-Deoxyadenosine, a major anoxic radiolysis product of adenine in DNA, is a substrate for *Escherichia coli* endonuclease IV. *Biochemistry* 33 7842–7847. 10.1021/bi00191a011 7516707

[B28] Ischenko A. A. Saparbaev M. K. (2002). Alternative nucleotide incision repair pathway for oxidative DNA damage. *Nature* 415 183–187. 10.1038/415183a 11805838

[B29] Ishchenko A. A. Ide H. Ramotar D. Nevinsky G. Saparbaev M. (2004). α-anomeric deoxynucleotides, anoxic products of ionizing radiation, are substrates for the endonuclease IV-type AP endonucleases. *Biochemistry* 43 15210–15216. 15568813 10.1021/bi049214+

[B30] Izumi T. Mitra S. (1998). Deletion analysis of human AP-endonuclease: minimum sequence required for the endonuclease activity. *Carcinogenesis* 19 525–527. 10.1093/carcin/19.3.525 9525290

[B31] Izumi T. Schein C. H. Oezguen N. Feng Y. Braun W. (2004). Effects of backbone contacts 3′ to the abasic site on the cleavage and the product binding by human apurinic/apyrimidinic endonuclease (APE1). *Biochemistry* 43 684–689. 10.1021/bi0346190 14730972

[B32] Kanazhevskaya L. Y. Koval V. V. Vorobjev Y. N. Fedorova O. S. (2012). Conformational dynamics of abasic DNA upon interactions with AP endonuclease 1 revealed by stopped-flow fluorescence analysis. *Biochemistry* 51 1306–1321. 10.1021/bi201444m 22243137

[B33] Kelley R. M. Georgiadis M. M. Fishel L. M. (2011). APE1/Ref-1role in redox signaling: translational applications of targeting the redox function of the DNA repair/redox protein APE1/Ref-1. *Curr. Mol. Pharmacol.* 5 36–53. 10.2174/1874467211205010036 22122463 PMC3319314

[B34] Kladova O. A. Bazlekowa-Karaban M. Baconnais S. Piétrement O. Ishchenko A. A. Matkarimov B. T. (2018a). The role of the N-terminal domain of human apurinic/apyrimidinic endonuclease 1, APE1, in DNA glycosylase stimulation. *DNA Repair (Amst).* 64 10–25. 10.1016/j.dnarep.2018.02.001 29475157

[B35] Kladova O. A. Grin I. R. Fedorova O. S. Kuznetsov N. A. Zharkov D. O. (2019). Conformational dynamics of damage processing by human DNA glycosylase NEIL1. *J. Mol. Biol.* 431 1098–1112. 10.1016/j.jmb.2019.01.030 30716333

[B36] Kladova O. A. Iakovlev D. A. Groisman R. Ishchenko A. A. Saparbaev M. K. Fedorova O. S. (2020). An assay for the activity of base excision repair enzymes in cellular extracts using fluorescent DNA probes. *Biochemistry (Moscow)* 85 480–489. 10.1134/S0006297920040082 32569555

[B37] Kladova O. A. Krasnoperov L. N. Kuznetsov N. A. Fedorova O. S. (2018b). Kinetics and thermodynamics of DNA processing by wild type DNA-glycosylase endo III and its catalytically inactive mutant forms. *Genes (Basel)* 9:190. 10.3390/genes9040190 29601551 PMC5924532

[B38] Kuzmic P. (1996). Program DYNAFIT for the analysis of enzyme kinetic data: application to HIV proteinase. *Anal. Biochem.* 237 260–273. 10.1006/abio.1996.0238 8660575

[B39] Kuznetsov N. A. Koval V. V. Zharkov D. O. Fedorova O. S. (2012a). Conformational dynamics of the interaction of *Escherichia coli* endonuclease VIII with DNA substrates. *DNA Repair* 11 884–891. 10.1016/j.dnarep.2012.08.004 23000248

[B40] Kuznetsov N. A. Kuznetsova A. A. Vorobjev Y. N. Krasnoperov L. N. Fedorova O. S. (2014). Thermodynamics of the DNA damage repair steps of human 8-oxoguanine DNA glycosylase. *PLoS One* 9:e98495. 10.1371/journal.pone.0098495 24911585 PMC4049573

[B41] Kuznetsov N. A. Vorobjev Y. N. Krasnoperov L. N. Fedorova O. S. (2012b). Thermodynamics of the multi-stage DNA lesion recognition and repair by formamidopyrimidine-DNA glycosylase using pyrrolocytosine fluorescence–stopped-flow pre-steady-state kinetics. *Nucleic Acids Res.* 40 7384–7392. 10.1093/nar/gks423 22584623 PMC3424566

[B42] Kuznetsova A. A. Fedorova O. S. Kuznetsov N. A. (2018a). Kinetic Features of 3′-5′ exonuclease activity of human AP-Endonuclease APE1. *Molecules* 23:2101. 10.3390/molecules23092101 30134601 PMC6225374

[B43] Kuznetsova A. A. Kuznetsov N. A. Ishchenko A. A. Saparbaev M. K. Fedorova O. S. (2014). Pre-steady-state fluorescence analysis of damaged DNA transfer from human DNA glycosylases to AP endonuclease APE1. *Biochim. Biophys. Acta* 1840 3042–3051. 10.1016/j.bbagen.2014.07.016 25086253

[B44] Kuznetsova A. A. Matveeva A. G. Milov A. D. Vorobjev Y. N. Dzuba S. A. Fedorova O. S. (2018b). Substrate specificity of human apurinic/apyrimidinic endonuclease APE1 in the nucleotide incision repair pathway. *Nucleic Acids Res.* 46 11454–11465. 10.1093/nar/gky912 30329131 PMC6265485

[B45] Kuznetsova A. A. Novopashina D. S. Fedorova O. S. Kuznetsov N. A. (2020). Effect of the substrate structure and metal ions on the hydrolysis of undamaged RNA by human AP endonuclease APE1. *Acta Nat.* 2 33–44. 10.32607/actanaturae.10864 PMC7385091 32742730

[B46] Li M. Wilson D. M. III (2014). Human apurinic/apyrimidinic endonuclease 1. *Antioxid Redox Signal* 20 678–707. 10.1089/ars.2013.5492 23834463 PMC3901322

[B47] Lipton A. S. Heck R. W. Primak S. McNeill D. R. Wilson D. M. III Ellis P. D. (2008). Characterization of Mg2+ binding to the DNA repair protein apurinic/apyrimidic endonuclease 1 via solid-state 25Mg NMR spectroscopy. *J. Am. Chem. Soc.* 130 9332–9341. 10.1021/ja0776881 18576638 PMC2564828

[B48] Liu T. C. Lin C. T. Chang K. C. Guo K. W. Wang S. Chu J. W. (2021). APE1 distinguishes DNA substrates in exonucleolytic cleavage by induced space-filling. *Nat. Commun.* 12:601. 10.1038/s41467-020-20853-2 33504804 PMC7841161

[B49] Manvilla B. A. Pozharski E. Toth E. A. Drohat A. C. (2013). Structure of human apurinic/apyrimidinic endonuclease 1 with the essential Mg2+ cofactor. *Acta Crystallogr. D Biol. Crystallogr.* 69 2555–2562. 10.1107/S0907444913027042 24311596 PMC3852660

[B50] Masuda Y. Bennett R. A. Demple B. (1998). Rapid dissociation of human apurinic endonuclease (Ape1) from incised DNA induced by magnesium. *J. Biol. Chem.* 273 30360–30365. 9804799 10.1074/jbc.273.46.30360

[B51] Meira L. B. Devaraj S. Kisby G. E. Burns D. K. Daniel R. L. Hammer R. E. (2001). Heterozygosity for the mouse Apex gene results in phenotypes associated with oxidative stress. *Cancer Res.* 61 5552–5557. 11454706

[B52] Miroshnikova A. D. Kuznetsova A. A. Kuznetsov N. A. Fedorova O. S. (2016a). Thermodynamics of damaged DNA binding and catalysis by human AP endonuclease 1. *Acta Nat.* 8 103–110. PMC4837577 27099790

[B53] Miroshnikova A. D. Kuznetsova A. A. Vorobjev Y. N. Kuznetsov N. A. Fedorova O. S. (2016b). Effects of mono- and divalent metal ions on DNA binding and catalysis of human apurinic/apyrimidinic endonuclease 1. *Mol. BioSyst.* 12 1527–1539. 10.1039/c6mb00128a 27063150

[B54] Mol C. D. Hosfield D. J. Tainer J. A. (2000a). Abasic site recognition by two apurinic/apyrimidinic endonuclease families in DNA base excision repair: the 3′ ends justify the means. *Mutat. Res.* 460 211–229. 10946230 10.1016/s0921-8777(00)00028-8

[B55] Mol C. D. Izumi T. Mitra S. Tainer J. A. (2000b). DNA-bound structures and mutants reveal abasic DNA binding by APE1 and DNA repair coordination. *Nature* 403 451–456. 10.1038/35000249 10667800

[B56] Moor N. Vasil’eva I. Lavrik O. (2020). Functional role of N-terminal extension of human ap endonuclease 1 in coordination of base excision dna repair via protein–protein interactions. *Int. J. Mol. Sci.* 21:3122. 10.3390/ijms21093122 32354179 PMC7247576

[B57] Oezguen N. Schein C. H. Peddi S. R. Power T. D. Izumi T. Braun W. (2007). A “moving metal mechanism” for substrate cleavage by the DNA repair endonuclease APE-1. *Proteins* 68 313–323. 10.1002/prot.21397 17427952

[B58] Poletto M. Vascotto C. Scognamiglio P. L. Lirussi L. Marascod D. Tell G. (2013). Role of the unstructured N-terminal domain of the hAPE1 (human apurinic/apyrimidinic endonuclease 1) in the modulation of its interaction with nucleic acids and NPM1 (nucleophosmin). *Biochem. J.* 452 545–557. 10.1042/BJ20121277 23544830

[B59] Popov A. V. Grin I. R. Dvornikova A. P. Matkarimov B. T. Groisman R. Saparbaev M. (2020). Reading targeted DNA damage in the active demethylation pathway: role of accessory domains of eukaryotic AP endonucleases and thymine-DNA glycosylases. *J. Mol. Biol.* 10.1016/j.jmb.2019.12.020 [Epub ahead of print], 31866293

[B60] Prorok P. Alili D. Saint-Pierre C. Gasparutto D. Zharkov D. O. Ishchenko A. A. (2013). Uracil in duplex DNA is a substrate for the nucleotide incision repair pathway in human cells. *Proc. Natl. Acad. Sci. U.S.A.* 110 E3695–E3703. 10.1073/pnas.1305624110 24023064 PMC3785768

[B61] Shen J. C. Loeb L. A. (2003). Mutations in the α8 loop of human APE1 alter binding and cleavage of DNA containing an abasic site. *J. Biol. Chem.* 278 46994–47001. 10.1074/jbc.M309362200 12966083

[B62] Tell G. Wilson D. M. Lee C. H. (2010). Intrusion of a DNA repair protein in the RNome world: is this the beginning of a new era? *Mol. Cell. Biol.* 30 366–371. 10.1128/mcb.01174-09 19901076 PMC2798471

[B63] Timofeyeva N. A. Fedorova O. S. (2016). A kinetic mechanism of repair of DNA containing alpha-anomeric deoxyadenosine by human apurinic/apyrimidinic endonuclease 1. *Mol. Biosyst.* 12 3435–3446. 10.1039/c6mb00511j 27722620

[B64] Timofeyeva N. A. Koval V. V. Ishchenko A. A. Saparbaev M. K. Fedorova O. S. (2011). Kinetic mechanism of human apurinic/apyrimidinic endonuclease action in nucleotide incision repair. *Biochemistry* 76 273–281. 21568862 10.1134/s0006297911020155

[B65] Timofeyeva N. A. Koval V. V. Knorre D. G. Zharkov D. O. Saparbaev M. K. Ishchenko A. A. (2009). Conformational dynamics of human AP endonuclease in base excision and nucleotide incision repair pathways. *J. Biomol. Struct. Dyn.* 26 637–652. 10.1080/07391102.2009.10507278 19236113

[B66] Toseland C. P. Webb M. R. (2013). ATPase mechanism of the 5′-3′ DNA helicase, RecD2. *J. Biol. Chem.* 288 25183–25193. 10.1074/jbc.M113.484667 23839989 PMC3757182

[B67] Tsutakawa S. E. Shin D. S. Mol C. D. Izumi T. Arvai A. S. Mantha A. K. (2013). Conserved structural chemistry for incision activity in structurally non-homologous apurinic/apyrimidinic endonuclease APE1 and endonuclease IV DNA repair enzymes. *J. Biol. Chem.* 288 8445–8455. 23355472 10.1074/jbc.M112.422774 PMC3605660

[B68] Unnikrishnan A. Raffoul J. J. Patel H. V. Prychitko T. M. Anyangwe N. Meira L. B. (2009). Oxidative stress alters base excision repair pathway and increases apoptotic response in apurinic/apyrimidinic endonuclease 1/redox factor-1 haploinsufficient mice. *Free Radic. Biol. Med.* 46 1488–1499. 10.1016/j.freeradbiomed.2009.02.021 19268524 PMC2677124

[B69] Vascotto C. Fantini D. Romanello M. Cesaratto L. Deganuto M. Leonardi A. (2009). APE1/Ref-1 Interacts with NPM1 within nucleoli and plays a role in the rRNA quality control process. *Mol. Cell. Biol.* 29 1834–1854. 10.1128/MCB.01337-08 19188445 PMC2655621

[B70] Wong D. DeMott M. S. Demple B. (2003). Modulation of the 3′→5′-exonuclease activity of human apurinic endonuclease (Ape1) by its 5′-incised abasic DNA product. *J. Biol. Chem.* 278 36242–36249. 10.1074/jbc.M306065200 12857737

[B71] Xanthoudakis S. (1996). The redox/DNA repair protein, Ref-1, is essential for early embryonic development in mice. *Proc. Natl. Acad. Sci. U.S.A.* 93 8919–8923. 10.1073/Pnas.93.17.8919 8799128 PMC38569

